# Two-stage sampling in the estimation of growth parameters and percentile norms: sample weights versus auxiliary variable estimation

**DOI:** 10.1186/s12874-021-01353-3

**Published:** 2021-08-17

**Authors:** George Vamvakas, Courtenay Norbury, Andrew Pickles

**Affiliations:** 1 Department of Biostatistics and Health Informatics, Institute of Psychology, Psychiatry and Neuroscience, Kings College London, London, UK; 2 Psychology and Language Sciences, University College London, London, UK

**Keywords:** Auxiliary variable, Weights, Two-stage design, Missing data, Population norms, Percentile norms

## Abstract

**Background:**

The use of auxiliary variables with maximum likelihood parameter estimation for surveys that miss data by design is not a widespread approach, despite its documented improved efficiency over traditional approaches that deploy sampling weights. Although efficiency gains from the use of Normally distributed auxiliary variables in a model have been recorded in the literature, little is known about the effects of non-Normal auxiliary variables in the parameter estimation.

**Methods:**

We simulate growth data to mimic SCALES, a two-stage survey of language development with a screening phase (stage one) for which data are observed for the whole sample and an intensive assessments phase (stage two), for which data are observed for a sub-sample, selected using stratified random sampling. In the simulation, we allow a fully observed Poisson distributed stratification criterion to be correlated with the partially observed model responses and develop five generalised structural equation growth models that host the auxiliary information from this criterion. We compare these models with each other and with a weighted growth model in terms of bias, efficiency, and coverage. We finally apply our best performing model to SCALES data and show how to obtain growth parameters and population norms.

**Results:**

Parameter estimation from a model that incorporates a non-Normal auxiliary variable is unbiased and more efficient than its weighted counterpart. The auxiliary variable method is capable of producing efficient population percentile norms and velocities.

**Conclusions:**

The deployment of a fully observed variable that dominates the selection of the sample and correlates strongly with the incomplete variable of interest appears beneficial for the estimation process.

## Content

Text and results for this section, as per the individual journal’s instructions for authors.

## Background

The topic of children’s growth has been attracting the attention of health professionals for almost a century. Reports and studies of anthropometric measurements appeared initially in the U.S.A. in the early 1930s and included information on height, weight, subcutaneous tissue folds and several body parts. These measurements formed part of the application of preventative medicine, as health practitioners started to recognise its usefulness towards the link between malnutrition and stunted growth [1]. However, to assess whether a child was developing as expected, this information alone was not sufficient. What was needed was the establishment of benchmarks that would allow a comparison of a child’s measurements against what was considered to be normal. This would enable practitioners to develop an understanding of whether the growth of an individual tracked that of healthy peers. Creation of benchmarks or norms, therefore, became topical among health practitioners at first and subsequently among statisticians.

Norms are summary statistics of measurements, commonly calculated using simple random samples of typically developing children, who form the reference population. These summary statistics appear, for example, as standard scores or as percentiles and are usually presented for several ages, so that age-specific comparisons can be made between a child and the reference population. Typically, norms are presented in the form of a graph known as a growth chart.

Growth can be assessed via three tools. Each one answers qualitatively different questions. The first tool refers to what is frequently termed as distance standards. Distance standards gauge the status of a person’s growth at a particular point in time. Their construction requires cross-sectional information across ages (longitudinal information that is treated as cross-sectional can also be used [2]). According to Tanner [1], two of the best early examples of distance norms were the works by Gore and Palmer in 1949 and by Daley in 1950, who presented standards for height and weight for British children. The second and third tools are termed velocity and acceleration standards. They are mentioned together because they both require longitudinal data to be estimated. They answer questions about how fast or slow a person grows during a particular period (velocity standards), and whether a person’s growth during a period is accelerated or decelerated when compared to their growth during another period (acceleration standards).

In this paper we will develop and evaluate via simulation a model for the estimation of growth parameters for two-stage sample designs and will show how we can manipulate these parameters to obtain distance percentile norms for language. We will show that our model has the potential to answer questions relating to the normality of a child’s attained language skills such as, what is a child’s current language ability, what can a child’s expected language development be and what is the speed with which a child is improving in these skills compared to their peers. In a previous paper ([2]) we produced Z-scores and distance percentile curves for six language indices. There, we used a method known as the LMS method [3] and produced population scores and percentiles after embedding weights in the estimation process. LMS assumes the raw scale scores at a given time are approximately standard normal after application of a skew-removing transformation. This method is capable of simultaneous estimation of centile-curves at different ages and is arguably one of the most popular transformation method due to its efficient use of data and flexibility [4–9].

As in [2], this paper too, will utilise data from the SCALES study (see [10, 11] for details). SCALES is an ongoing two-stage (screening/in-depth assessment) population longitudinal survey of UK children attending mainstream reception classrooms. It comprises a unique sample with a wider range of language and non-verbal cognitive profiles than those reported in previous studies of language disorder (e.g. Beitchman et al. [12]; Tomblin et al. [13]) and more in-depth language phenotyping relative to existing cohort studies (e.g. Avon Longitudinal Study of Parents and Children, Millennium Cohort Study).

### Weights

The estimation of population parameters from stratified surveys has often been approached by weighting. Weights can be functions of probabilities, measurement precisions, and sample sizes. In its simplest form, a weight for subject *i* is calculated as the inverse of their selection probability *p*_*i*_. Weights can compensate not only for observations which were never planned to be collected (missing by design), but also for observations which were planned to be collected but were not (missing due to non-response).

Pfeffermann [14, 15] asserts that weights can safeguard the analyst against *non-ignorable* sampling designs and model mis-specifications. Non-ignorability refers to the situation where information on how the sample was selected cannot be ignored during the inferential process. When omissions of important interactions or mis-specifications of the relationship between the response and the explanatory variables exist, use of weights can help to correct the estimated results (see [16] and [17]).

Despite these advantages, the use of weights has limitations. The major limitation is that probability-weighted estimators are generally inefficient compared to unweighted estimators [16–19]. Pfeffermann [15] and Kalton [20] point out that an increase in variance and hence a decrease in the precision of the estimates is higher with smaller sample sizes and larger weight variability. When DeMets and Halperin [21] compared maximum likelihood (ML) with probability-weighted estimators under two simple stratified designs, they found that the ML estimators were considerably more efficient. Other limitations include the restriction of inference mainly to populations from which the sample was drawn, and the inability to conduct standard inferential procedures, such as the *likelihood ratio test* [15].

### Auxiliary information

Rubin [22] proves that when the data are missing at random (MAR), inference (frequentist and Bayesian) can proceed based only on what is observed, *ignoring* the ‘missingness’ mechanism. If the variable of interest is an incomplete variable *X*_*K*_, ignorability holds only when inference is based on the conditional distribution of *X*_*K*_|*X*_1_,...,*X*_*K*−1_, where *X*_1_,...,*X*_*K*−1_ are assumed to be fully observed variables associated with ‘missingness’ in *X*_*K*_, and the missing data mechanism *R* is independent of the partially complete data [15]. This requires that all variables affecting the selection process be identified and known for the whole target population [23]. An influential paper by Collins et al. [24] categorise these variables, which are referred to as *auxiliary* variables (AVs), into three classes, and maintain that their incorporation in the analysis yields unbiased results and increases in efficiency and statistical power. The first class involves variables correlated both with the partially observed variable *X*_*K*_ and the missingness mechanism *R*. The second class comprises variables that correlate with *X*_*K*_ but not with *R*. Finally, in the third class the authors list variables that are not correlated with *X*_*K*_, but may or may not be correlated with *R*. Their simulations suggest that omission of class one variables causes “substantial problems with bias, efficiency and coverage”. This, however, was evident only when the amount of missing data was quite large (over 50%) or when the correlation of the AVs with *X*_*K*_ or *R* was less than 0.40. Collins et al. showed that including in the model the class two variables can be very helpful for estimation, in terms of bias and “can add information that results in a decrease in standard errors”. They also argue that inclusion of class three variables has at worst no effect and at best an extremely beneficial effect on inference.

Savalei and Bentler [25] provide an intuitive explanation of why the incorporation of such correlated variables can be beneficial to estimation. They argue that a variable that is perfectly correlated with an incomplete variable offers perfect knowledge of the missing values and therefore, results are as efficient as those from fully observed analyses. Having said that, the authors warn against the use of a large number of AVs in a model and posit that doing so, might “add noise” to the model, potentially reversing any efficiency gains. It has been argued, however, that the effect of this is not severe; as White et al. [26] and Collins et al. [24] point out within the context of multiple imputation, loss of precision from model overfitting is often small and of no concern.

While the incorporation of AVs during a multiple imputation process is straightforward (see [27]), less clarity exists about how to utilise auxiliary information under ML. Graham [28] is one of few authors who shows how to include AVs when ML is the estimation method of choice. He specified two models for use within the structural equation modelling (SEM) framework. In an analogous way to multiple imputation, where the AVs are inserted into the imputation step leaving the substantive hypothesis of the analysis model intact, employment of latent-variable modelling too, allows for the incorporation of auxiliary information in the modelling process without altering the meaning of the substantive estimates. This means that inference does not have to rely on the conditional distribution *f*(*X*_*K*_|*X*_1_,...,*X*_*K*−1_) mentioned above leading some authors ([25, 29]) to define AVs as variables “unrelated to the substantive hypothesis”, carrying nonetheless information about the missing values or even the probability of being missing.

Graham [28] tests 3 models; a basic model, whose data are preprocessed by the expectation-maximisation (EM) algorithm, and two SEM models that incorporate AVs in different ways to each other. His simulations suggest that all three models yield very similar results, and insofar as the EM algorithm is equivalent to multiple imputation (the two methods produce identical results under certain mild conditions [24]) the two SEM models are very promising in terms of bias and efficiency. Graham used fully observed AVs. Enders [29] replicated Graham’s models allowing for missing data in the AVs, and found that although the partially observed AVs do not confer as big a benefit as their fully observed counterparts, it was clearly better to include them than to omit. Savalei and Bentler [25] presented an alternative way to incorporate AVs within a ML process based on an improved two-stage estimator, which overcomes convergence problems that may occur when the number of parameters in a model is large (such as with Graham’s model specification). Although their method performs similar to Graham’s in terms of bias and efficiency, it becomes slightly less efficient when the number of missing values in the dataset increases or when the data are MAR.

Auxiliary variables need not be Normally distributed. In the context of the SCALES study, for example, the design variable was an inexpensive and easy to use non-Normally distributed indicator of language difficulties. Despite this, not much research exists within the ML framework on whether AVs that are not Normally distributed have the same effect on bias and efficiency as those that are Normally distributed. Originally, the impact of the AVs on estimation was tested assuming some form of multi-variate Normality. DeMets and Halperin [21] presented estimators for multiple linear regression parameters assuming joint Normality. Holt et al. [30] produced Normally distributed AVs for inclusion in their ML model before they compared the OLS, ML, and probability weighted estimators. Nathan and Holt [31] relaxed the assumption of Normality imposed by DeMets and Halperin, and showed that consistent estimation can also be achieved under some milder restrictions, as long as auxiliary information is fully observed for the target population. Several more recent research studies (Collins et al. [24], Graham [28], Enders [29] and Savalei and Bentler [25]) deploy Normally distributed AVs in their multiple imputation or ML-based modelling comparisons.

## Simulation

The simulation study explores the impact of a discrete auxiliary variable on the bias and efficiency of ML estimators and compares them with a weighted estimator.

Fitted to datasets with incomplete data, different specifications of structural equation models that host auxiliary information were compared with each other and with a regression-based growth model that employed weights. A growth model fitted to the complete data, prior to the creation of missing data, served as the reference model.

The aim is the establishment of a model that handles auxiliary information adequately and yields estimates with desirable properties in a two-phase stratifying random sampling setting, analogous to the design of SCALES.

### The SCALES study and data generation

The data generation process was based on the SCALES study (see [32]) for which the consent procedures and the study protocol were developed in consultation with Surrey County Council and approved by the Royal Holloway Ethics Committee (where the study was initiated) in Year 1 of the study and the UCL Research Ethics Committee (9733/002) in Year 6 of the study. Informed consent was collected from parents/guardians before in-depth assessments in Year 1 and Year 6. Informed assent was collected from children prior to each assessment. Children were given certificates and small prizes at the end of each assessment session.

The SCALES cohort was selected using stratified random sampling. The study involved a two-phase design: in the first phase, screening data from 7,267 children were obtained. In this paper, we include monolingual English-speaking children only who attended mainstream schools and had phrased speech, bringing the total population of children down to 6,411. In the second phase, a sub-sample of 636 children were randomly selected for intensive individual assessment. Selection was based mainly on the Communication Checklist-Short (CCC-S) test, which is described in the Application section.

Scores from the intensive assessments were collected over three separate points in time; 490 children aged 5–6 years provided data during the 1^*st*^ year at school, 462 children aged 7–8 years provided data during the 3^*rd*^ year at school, and 337 children aged 10–11 years provided data during the 6^*th*^ year at school.

The parameters that would be used in the simulation to generate the data extracted from an unweighted growth model fitted to SCALES data. The model

1 $$\begin{array}{@{}rcl@{}} y_{ij}=\beta_{0}+\beta_{i0}+\beta_{1}age_{ij}+\beta_{i1}age_{ij}+\epsilon_{ij} \end{array} $$

provided estimates for the fixed intercept *β*_0_, fixed coefficient of age *β*_1_, random intercepts *β*_*i*0_, random coefficients *β*_*i*1_, residual term *ε*_*ij*_, and the covariance *ψ*_*ij*_ between *β*_*i*0_ and *β*_*i*1_. The outcome *y*_*ij*_ for participant *i* at occasion *j*, are repeated measurements from the Expressive One Word Picture Vocabulary Test, described in the Application section. The model used an unstructured random-effects covariance matrix and age was centred using the mean of the children’s age at each time point.

Simulated data for 2,000 participants were generated. Each participant had their own random intercept and coefficient, drawn from a bivariate Normal distribution with a mean of 0 and covariance matrix parameters equal to *β*_*i*0_,*β*_*i*1_, and *ψ*_*ij*_. It was assumed that participants had the same covariance parameters across all occasions. To construct the initial age variable, Normal variates were drawn using the marginal mean and standard deviation of the children’s ages at the first time point. For future ages, the average age difference between a subsequent time point and the first time point was added to the child’s age at time one. Having sampled values for the age variable, the random intercepts and the random coefficients, and using the estimated coefficients of the fixed intercept and fixed age from model 1 and an error term *e*_*ij*_∼*N*(0,*sd*(*ε*_*ij*_)), three outcome data per participant, one for each time point, were generated.

A variable that correlated with the individual random intercept *β*_*i*0_ was generated to act as our auxiliary variable. This variable followed a Poisson distribution, such that *PAux*∼*Po*(*β*_*i*0_/14). A rate of *β*_*i*0_/14 was selected after comparing the two distributions graphically, so that the shape of the distribution of *PAux* resembles the shape of the distribution of the design variable *CCC*−*S* from SCALES. The reason why this was done through graphical means is because we generally do not know the relationship between the auxiliary variable and the random intercept that is used in the calculation of the rate of the auxiliary variable. MAR missing data were created, conditioning on *PAux*; firstly, the probability of missingness was calculated as: *P*_*i*_(*miss*)=*exp*(−0.5+0.3∗*PAux*_*i*_)/(1+*exp*(−0.5+0.3∗*PAux*_*i*_)). Then, a uniformly distributed variable *U*_*i*_ was generated to help to create a missing value indicator coded 0 if *U*_*i*_>*PAux*_*i*_ and 1 if *U*_*i*_<*PAux*_*i*_. Values were deleted in *y*_*ij*_ and *age*_*ij*_ whenever the indicator was 0. The process produced around 50% of missing data. Weights were calculated by inverting *P*_*i*_(*miss*).

### Methods

We report on the ability of six models to recover the population parameter estimates. First, model 1 was fitted to the whole population (complete data model) assuming it is the correct population model and used its estimates as a benchmark to compare the estimates of alternative models. After creating missing data in the outcome and the age, model 1 was fitted again. We refer to this model as the naive model. A weighted version of model 1 (the weighted model), and a series of latent-variable growth models that included the auxiliary variable *PAux* were also fitted to the incomplete datasets. The latent-variable models allowed the fully observed *PAux* to be predicted by *β*_0*i*_, the growth parameter that describes the initial state of participant *i*.

The system of equations below shows the specification of the first latent-variable model, termed Poisson auxiliary model.

2 $$\begin{array}{@{}rcl@{}} y_{ij}&=&\beta_{0}+\beta_{i0}+\beta_{1}age_{ij}+\beta_{i1}age_{ij}+\epsilon_{ij1}  \\ \mu_{i}&=&\exp(\gamma_{0}+z_{1}\beta_{i0})  \\ \beta_{i1}&=&z_{2}\beta_{i0}+\epsilon_{i2}  \end{array} $$

where *PAux*_*i*_∼*Poisson*(*μ*_*i*_) is a time-invariant variable. It can be seen the auxiliary variable is used as the expected count outcome *μ*_*i*_ in a Poisson regression conditional on a constant and the random intercept. This setup respects the distribution of the auxiliary variable and the association between *PAux*_*i*_ and *β*_0*i*_ imposed during the data generation. The association between the random slope and random intercept entered the model in the form of a simple linear regression of *β*_1*i*_ on *β*_0*i*_. This equation did not include a constant, so that *β*_1*i*_ was proportional to *β*_0*i*_.

The second structural equation model was identical to model 2 except that now the auxiliary variable was transformed using a skew-removing function (see Software section) and treated as a continuous outcome in a simple Gaussian model equation. We refer to this model as the transformed Poisson auxiliary model.

The next model specification introduces an extra equation in which the outcome is a binary indicator coded 1 if *y*_*ij*_ is observed and 0 otherwise. In this setup, the model, termed Poisson/missingness model, allowed the random intercept to affect not only *PAux*_*i*_, but also the probability of missingness *P*_*i*_(*miss*). This took the form:

3 $$\begin{array}{@{}rcl@{}} y_{ij}&=&\beta_{0}+\beta_{i0}+\beta_{1}age_{ij}+\beta_{i1}age_{ij}+\epsilon_{ij1}  \\ \mu_{i}&=&\exp(\gamma_{0}+z_{1}\beta_{i0}) \\ \beta_{i1}&=&z_{2}\beta_{i0}+\epsilon_{i2}  \\ log\left[\frac{P_{i}(miss)}{1-P_{i}(miss)}\right]&=&\delta_{0}+z_{3}\beta_{i0}  \end{array} $$

where now *P*_*i*_(*miss*) forms the log-odds of a missing observation in a logistic regression model which includes the random intercept and a constant.

As with the Poisson auxiliary model, a different version of the Poisson/missingness model was trialled after transforming the auxiliary variable using the same skew-removing function and replacing the Poisson equation in (3) with a Gaussian equation. We call this model the transformed Poisson/missingness model.

Finally, we revisit model 2 but this time we allow our count auxiliary variable to be modelled alongside an over-dispersion parameter by treating it as the outcome of a negative binomial regression.

### Estimands

The target of the simulations were five estimands; the fixed intercept *β*_0_, the fixed effect of age *β*_1_, the variances of *β*_0*i*_ and *β*_1*i*_, and the covariance between *β*_0*i*_ and *β*_1*i*_. According to Rubin [33], an estimand is defined as “the quantity of scientific interest that can be calculated in the population and does not change its value depending on the data collection design used to measure it”. For these simulated datasets, *β*_0_ represents the average value of the outcome measure for subjects at mean age who share similar unobserved growth characteristics. *β*_1_ is the fixed effect of age, conditional on the unobserved growth characteristics. The latent quantities *β*_0*i*_ and *β*_1*i*_ represent variability in unobserved characteristics of growth; *β*_0*i*_ refers to the initial state of individual *i*, and *β*_1*i*_ to a latent growth trajectory across the span of the observation time.

### Performance measures

The performance of the estimators targeting the estimands was assessed primarily by three measures: the bias, the empirical standard deviation, and the coverage of the estimator.

The bias of an estimator quantifies the average proximity of a parameter estimate to the population value (estimand) upon repeated sampling. For any tested parameter, the empirical estimate of bias is obtained as: $\frac {1}{N_{sims}}\sum _{h=1}^{N_{sims}}\hat {\theta }_{h}-\theta $, where $\hat {\theta }$ are estimates of the true parameter *θ* [34].

The empirical standard deviation (ESD) describes the precision or efficiency of the estimator of *θ* and is given by the standard deviation of $\hat {\theta }_{h}$ over *n*_*sims*_: $\sqrt {\frac {1}{N_{sims}-1}\sum _{h=1}^{N_{sims}}\left (\hat {\theta }_{h}-\bar {\theta }\right)^{2}}$, where $\bar {\theta }$ is the average of the parameter estimates $\hat {\theta }$ over the total number of simulation runs. As such, the ESD depends only on $\hat {\theta }$. For an estimator to be precise this measure is expected to be as small as possible. A low bound, however, is hard to establish, and for this reason the ESD of a model is reported relative to the ESD from the complete data model. As Morris et al. [34] show, this is calculated as:

4 $$\begin{array}{@{}rcl@{}} 100\left(\left(\frac{\widehat{ESD_{complete}}}{\widehat{ESD_{model_{m}}}}\right)^{2}-1\right), \end{array} $$

where $\widehat {ESD_{model}}$ is the estimated ESD of model *m*, and $\widehat {ESD_{complete}}$ the estimated ESD of the reference complete data model.

To investigate the precision of the estimators, we report the average model standard error, $\widehat {ModelSE}$, and its value relative to the model’s ESD:

5 $$\begin{array}{@{}rcl@{}} 100\left(\frac{\widehat{ModelSE}}{\widehat{ESD}}-1\right). \end{array} $$

The model standard error targets the empirical standard deviation and the former should on average be equal to the latter. A disagreement between the two quantities denotes bias in the estimation of the model standard error [34].

Generally, coverage is defined as the probability that a 100(1−*α*)*%* Confidence Interval (CI) contains the true parameter *theta*. Here, we evaluate whether the empirical coverage of each parameter approaches the nominal 95% coverage rate.

The performance measures are estimates themselves and therefore subject to uncertainty. For this reason, the Monte Carlo standard error, a measure that reflects the simulation uncertainty about these estimates, is shown next to each performance value. Efficiency and coverage rates were interpreted with bias born in mind. A complication in the interpretation of simulation results arises from the fact that bias can be a source of both small model standard errors and under-coverage. More specifically, Morris et al. [34] state that where the estimators are biased towards the null hypothesis the standard errors will be invariably smaller. The authors go on to explain that coverage rates are influenced by three things; i) bias, ii) the degree to which the model standard error agrees with the ESD, and iii) the sampling distribution of the estimated parameter. Bias not equal to zero results in under-coverage. Disagreement between the model standard error and the ESD results in under-coverage if *ModelSE*<*ESD*, and in over-coverage if *ModelSE*>*ESD*. A non-Normal sampling distribution of the parameter results in under-coverage.

### Software

One thousand simulated datasets were generated to test each model. This was carried out in Stata v.16. The rnormal(m,s) command was used to draw Normal deviates, and the rpoisson(m) command to simulate count data. The inverse logit function (expit) invlogit() was used for *P*_*i*_(*miss*) and the runiform() for *U*_*i*_. *PAux* was transformed using lnskew0 which attempts to create a Normally distributed variable with 0 skewness using the Box-Cox transformation [35].

All models were fitted using Stata’s suite for generalised structural equation models gsem. Although the Population, the Naive, and the Weighted models are regression-based models that can be fitted using multi-level model software we opted to fit these models with gsem to be consistent with the implementation of the latent variable models. With gsem the responses can be continuous or binary, ordinal, count, or multinomial. The package can handle a wide variety of models such as linear regression, (ordinal) logit, (ordinal) probit, Poisson, negative binomial, and multinomial logit. Models can be fitted to single- or multi-level data and latent variables can be included at any level. Models can have continuous latent variables or categorical latent variables but not both. gsem’s estimation method is maximum-likelihood. It includes four integration methods (see Stata’s sem manual for details) and where there are no closed analytical forms, integrals are approximated by adaptive quadrature [36].

All performance measures were generated by simsum, written by Ian White [37]. An attractive feature of this command is the calculation of the Monte Carlo error, which is often ignored in the reporting of simulation results.

## Simulation results

The convergence rate was 100% for all models except for the negative binomial auxiliary model, which converged in 677 simulation runs; since the simulated data had no over-dispersion for which the corresponding negative binomial over-dispersion parameter would be minus infinite, we would expect estimation problems in 50% of samples. In fact, the convergence rate for this model was 67.7%.

Subsequent analyses include converged models only. In what follows, all models except for the complete data model are sometimes collectively called the *missing data* models.

Table 1 presents the bias along with the corresponding Monte Carlo standard error of the simulation, by model and by parameter. The existence of significant biases is highlighted in red; deep red denotes bias values that are 50% or more of the ESD value from the complete data model for the specific parameter. Cells in light red, denote biases that lie between 20% and 50% of the ESD value from the complete data model. Table 2 displays the percentage increase (or decrease) in a model’s precision relative to the precision of the parameter from the complete data model, calculated using formula 4. Values highlighted in red show which model returned the largest precision discrepancy for each parameter. Table 3 displays the results from a comparison between a model’s average standard error for an estimate and its corresponding ESD value, based on formula 5. Finally, Table 4 displays the coverage rates by model and parameter. Values highlighted in red represent some of the lowest rates observed in the table.

**Table 1:**
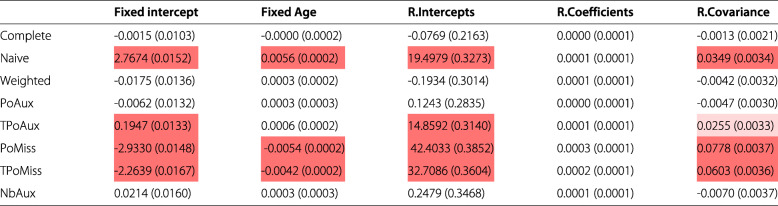
Bias (and Monte Carlo standard error) of parameters by model

Values highlighted in red denote significant biases, as defined in the text. The models are abbreviated as follows: *Complete* the complete data model, *Naive* model 1 fitted to incomplete data, *Weighted* the Weighted model, *PoAux* the Poisson auxiliary model, *TPoAux* the transformed Poisson auxiliary model, *PoMiss* the Poisson/missingness model, *TPoMiss* the transformed Poisson/missingness model, *NbAux* the negative binomial auxiliary model

**Table 2:**
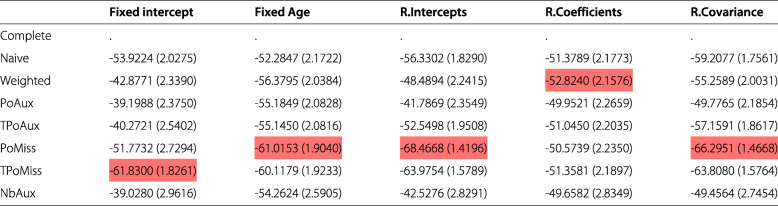
% increase (decrease) in precision (and Monte Carlo standard error) relative to the complete data model

Values highlighted in red indicate the largest difference from the complete data model by parameter. The models are abbreviated as follows: *Complete* the complete data model, *Naive* model 1 fitted to incomplete data, *Weighted* the Weighted model, *PoAux* the Poisson auxiliary model, *TPoAux* the transformed Poisson auxiliary model, *PoMiss* the Poisson/missingness model, *TPoMiss* the transformed Poisson/missingness model, *NbAux* the negative binomial auxiliary model

**Table 3:** Average model standard error (and Monte Carlo standard error) relative to empirical standard deviation

	Fixed intercept	Fixed Age	R.Intercepts	R.Coefficients	R.Covariance
Complete	0.5694 (2.2505)	-0.4001 (2.2288)	-0.6407 (2.2251)	-1.6948 (2.2007)	3.3954 (2.3145)
Naive	2.1402 (2.2864)	-1.3941 (2.2074)	2.6297 (2.3006)	-1.6499 (2.2035)	-1.0186 (2.2171)
Weighted	8.5475 (2.4301)	-2.9089 (2.1738)	0.1789 (2.2503)	-0.4633 (2.2335)	-1.3902 (2.2119)
PoAux	-0.0666 (2.2366)	-3.0192 (2.1711)	-0.4454 (2.2310)	-1.1911 (2.2138)	-1.2034 (2.2128)
TPoAux	1.7493 (2.2775)	-3.1518 (2.1681)	-2.7690 (2.1791)	-1.5976 (2.2046)	-1.3179 (2.2104)
PoMiss	3.3083 (2.3130)	-4.2109 (2.1448)	1.2974 (2.2726)	-1.4011 (2.2091)	-1.1374 (2.2158)
TPoMiss	-3.3571 (2.1646)	-4.4398 (2.1396)	-4.8465 (2.1341)	-1.6610 (2.2033)	-1.5355 (2.2064)
NbAux	0.6920 (2.7424)	-1.9741 (2.6677)	-0.9206 (2.6994)	-0.6157 (2.7084)	-0.6002 (2.7096)

The higher the value in absolute terms the larger the discrepancy between the model standard error and the empirical standard deviation. The models are abbreviated as follows: *Complete* the complete data model, *Naive* model 1 fitted to incomplete data, *Weighted* the Weighted model, *PoAux* the Poisson auxiliary model, *TPoAux* the transformed Poisson auxiliary model, *PoMiss* the Poisson/missingness model, *TPoMiss* the transformed Poisson/missingness model, *NbAux* the negative binomial auxiliary model

**Table 4:**
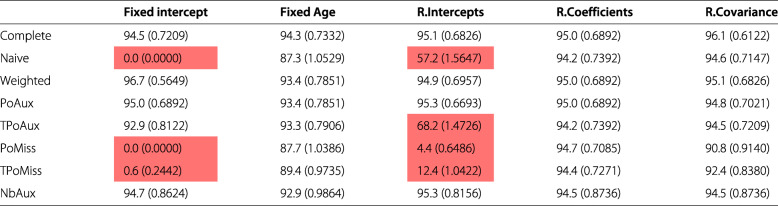
Coverage of model (and Monte Carlo standard error) by parameter

Values highlighted in red represent extremely low rates of coverage. The models are abbreviated as follows: *Complete* the complete data model, *Naive* model 1 fitted to incomplete data, *Weighted* the Weighted model, *PoAux* the Poisson auxiliary model, *TPoAux* the transformed Poisson auxiliary model, *PoMiss* the Poisson/missingness model, *TPoMiss* the transformed Poisson/missingness model, *NbAux* the negative binomial auxiliary model

**Fig. 1:**
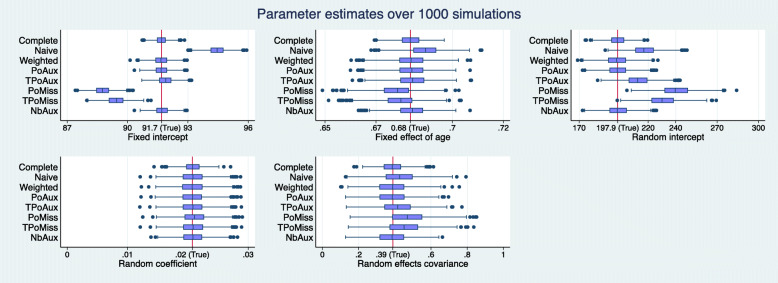
Bias: Box-plots of coefficient estimates over 1000 simulations. The vertical red line is positioned on the true population value. The line in the middle of each box represents the median estimate value over the total number of simulations

**Fig. 2:**
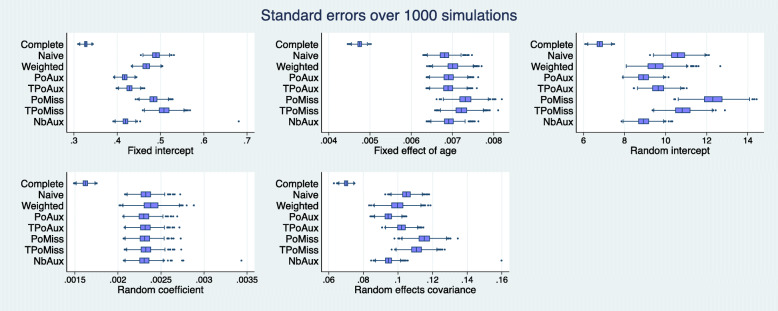
Precision: Box-plots of standard errors over 1000 simulations. The line in the middle of each box represents the median value of the standard errors over the total number of simulations

Figures 1 and 2 display the sampling distribution of $\hat {\theta }_{h}$ and $\widehat {SE}(\hat {\theta }_{h})$, respectively, for each one of the five estimands, as estimated by each model over 1000 simulation runs. The distributions appear symmetrical for all models and across all parameter estimates. The blue line inside the boxes show the median of the sampling distribution. The vertical red lines in the five panels of Fig. 1 represents the true population value.

As expected, the complete data model returns the least biased and most precise estimates across the models (Figs. 1 and 2). Its ESD values agree very closely with its average model standard errors with percentage differences that range between 0.4% and 3.4%, in absolute terms (Table 3). The complete data model consistently achieves coverage rates close to the nominal level for all parameters. These range from 94.3% to 96.1% (Table 4).

The Naive model over-estimates the true value of all parameters except that for the random coefficients (Fig. 1). All these biases are considerable and more than 50% of the ESD value of the complete data model, as highlighted in Table 1. The coverage for the model’s only unbiased parameter, the random coefficients, is 94.2% (Table 4).

The weighted model estimates all parameters without any significant bias (there are no highlighted values in Table 1). Among the missing data models the weighted model estimates the random coefficients parameter with the smallest precision (Fig. 2, random coefficients parameter). This does not appear to be the result of a bias in the estimation of the standard error, since the model’s standard error and the ESD value for this parameter are only 0.5 percentage points away from each other (Table 3), the smallest percentage difference among the missing data models. The ESD for the random coefficients is 52.8% less than that of the complete data model, which represents the largest discrepancy observed among the missing data models (Table 2). Table 3 shows that all average model standard errors for the weighted model are less than 3% of the corresponding ESD values, except that for the fixed intercept parameter which is 8.6% more than the corresponding ESD value. Again, this is the largest observed discrepancy among the missing data models and may explain the reason behind the somewhat elevated coverage rate seen for this parameter in Table 4. Despite this, the weighted model appears to cover all parameters well.

None of the parameters estimated by the Poisson auxiliary model appear biased (Table 1 and Fig. 1). An examination of the standard errors in Fig. 2 reveals that the Poisson auxiliary model displays some of the best behaviours. The model returns some of the most precise estimates among the missing data models; compared to the weighted model, in particular, it appears to be more efficient. This can be seen by inspecting Table 2: a look at the fixed intercept, for example, shows that the weighted model is 42.9% less efficient than the complete data model, whereas the Poisson auxiliary model is 39.2% less efficient than the complete data model, and this is the case for all parameters. Moreover, the model standard errors appear to be unbiased. The average standard errors and the ESD values appear to be very close to each other, with values close to 0 for all parameters except for the fixed-effect of age (the model standard error for this parameter is approximately 3.0% less than its ESD value, as seen in Table 3). This discrepancy, however, does not seem to be extreme relative to the rest of the models. The coverage rates achieved by the Poisson auxiliary model are close to the nominal level for all parameters.

The Box-Cox transformation of the Poisson auxiliary variable did not produce a perfectly Normal variable and hence the transformed auxiliary model was mis-specified. The model shows considerable bias for three out of the five parameters (Table 1). The estimates for the fixed and random intercepts are 50% larger than the complete data model’s corresponding ESD values, and the estimate for the random-effects covariance lies between 20 and 50% of the ESD value of the complete data model for the same parameter. The two unbiased parameters, i.e. the fixed-effect of age and the random coefficients, appear to be estimated efficiently with no discernible difference between the model’s SE and ESD values. Their coverage is acceptable, with a rate of 93.3% for the fixed-effect of age and 94.2% for the random-coefficients of age.

The Poisson/missingness model yields significant bias for all parameters except for the random coefficients of age. For the fixed intercept and random intercepts in particular the model yields some of the most extreme biases (Fig. 1). The precision of the random coefficients, the only unbiased parameter, appears to be in line with what is observed for the rest of the missing data models in Fig. 2, and there is no large discrepancy between the model’s standard error and its ESD value for this parameter. In terms of coverage, all parameters have very poor rates except for the random coefficients, which achieve a value of 94.7%.

As with the Poisson/missingness model, the transformed Poisson/missingness model yields significant biases for all parameters except for the random coefficients of age. For the fixed- and random-intercepts parameters, this model too, seems to completely miss the true population value, as it can be seen in Fig. 1. The coverage rates for all but the random-coefficients parameter are suboptimal (Table 4).

Based on the 677 simulation runs for which the negative binomial auxiliary model converged, the model does not appear to produce bias during the estimation of the parameters, as it can be seen in Table and Fig. 1. Table 2 shows how the percentage differences in ESD values between the negative binomial auxiliary and the complete data model are very similar to the differences observed between its closely related Poisson auxiliary model and the complete data model. Inspection of Table 3 reveals that the negative binomial model returns some of the smallest discrepancies between the average model standard error and the corresponding ESD values for a parameter. Compared to the Poisson auxiliary model, the negative binomial model appears to be faring better for the fixed-effect of age, random coefficients of age, and the random-effects covariance in relation to the values seen in Table 3. In terms of coverage, the model achieves acceptable rates for all parameters, similar to those observed for the complete data, weighted, and Poisson auxiliary models.

## Application

We saw that specifying a generalised structural equation growth model that includes an extra equation in which a Poisson distributed auxiliary response is predicted by a random intercept, produces, after allowing the intercept factor to be associated with the slope factor, unbiased and more efficient parameters than its weighted counterpart. In this section, we fit such a model to SCALES data and explore its ability to produce population norms and centile curves analogous to those constructed by the LMS method in Vamvakas et al. (2019) [2]. We report and comment on the estimated growth parameters such as, the current language ability (predicted random intercepts) and the individual velocities (predicted random coefficients). We illustrate the methods and diagnostics using the Expressive One Word Picture Vocabulary (Expressive Vocabulary) test, and produce charts for the Expressive Vocabulary and the Narrative Recall tests. The input dataset contains information from the screening stage of SCALES and the three intensively assessed sub-populations. Our latent-variable model makes use of all four time-points (screening and three intensive assessments). We deploy graphical means to assess model fit and examine the efficiency of the norms via bootstrapping.

At the screening level, we retain information on the children’s age and gender, the number of children screened in each school and the CCC-S test scores. The CCC-S test is a brief version of the CCC-2 [38]. The CCC-2 has been shown to be highly effective at discriminating children with communication difficulties from typically developing children [38]. The CCC-S includes 13 items from the CCC-2 General Communication Composite. These items measure speech, morphology/syntax, semantics, and discourse skills in everyday contexts. For SCALES, teachers were requested to assess how often a range of language behaviours occur on a 4-point scale: 0 = rarely/never, 1 = occasionally, 2 = regularly; 3 = frequently/always. The CCC-S ranges from 0 to 39 with higher scores denoting worse outcomes.

At the intensive assessment level, we retain information on scores from the Expressive Vocabulary and the Narrative Recall tests and child age. During the Expressive Vocabulary test, the child is required to look at pictures and name concepts, objects or actions. The scores on the Expressive Vocabulary test range from 0 to 190 with higher values indicating better outcomes. The Cronbach’s Coefficient Alpha value, a measure of internal consistency, for children aged 5- to 8- years, is between 0.94 and 0.97. Test-retest reliability coefficients for the Expressive Vocabulary is also very high with values of 0.98 when raw scores are considered and 0.97 when standard scores are considered [39]. For the Narrative Recall test [40]) children are required to listen to a pre-recorded story about a monkey in a forest, which is played over headphones and is accompanied by pictures shown on a laptop screen. At the end of the story, the child is required to narrate it back in their own words and is given a mark for each correctly re-told part. The child’s retelling of the story is audio recorded. Narrative Recall ranges from 0 to 35 with higher scores indicating a better outcome. The Cronbach’s Coefficient Alpha value for children aged 6- to 11-years is 0.73.

Firstly, we fit the Poisson auxiliary model to scores from the Expressive Vocabulary test and compare its output with that from the weighted version of model 1. Sampling weights for the weighted model were estimated by inverting the predicted probability of the logistic model that included the variables that took part in the selection of the sample: *log*[*p*(*EV*_*i*_=1|*S*_*i*_,*N*_*i*_,*G*_*i*_)/(1−*p*(*EV*_*i*_=1|*S*_*i*_,*N*_*i*_,*G*_*i*_))]=*α*+*β*_1_*S*_*i*_+*β*_2_*N*_*i*_+*β*_3_*G*_*i*_, where *EV*_*i*_ is a binary indicator denoting whether scores from the Expressive Vocabulary test at school-year 3 are observed (coded 1) or not (coded 0), *S*_*i*_ is a binary indicator of communication difficulties based on the CCC-S test, *N*_*i*_ is the total number of pupils screened in a school, and *G*_*i*_ is an indicator for gender.

The Box-Cox transformation (*Y*^*λ*^−1)/*λ* was used to transform the Expressive Vocabulary responses throughout. The skewness minimisation parameter *λ* was chosen via maximum likelihood and/or via running model diagnostics. The ages at screen, and at the three school years were centred around the mean age of school year one. Goodness-of-fit was assessed by plotting the distributions of the level-1 residuals and the predicted values of the random intercepts and random slopes. We tested for overdispersion in the CCC-S using the BIC values from two models that used the screening variable either in a Poisson regression or in a negative binomial regression. As an additional test of model fit, we produced Z-scores from model parameters and compared these scores with those returned by the LMS method. The Z-scores were estimated as:

6 $$\begin{array}{@{}rcl@{}} Z_{ji}=\frac{y_{ji}-\mathbf{X}_{i}\mathbf{b}}{\sqrt{var(y_{ji}|\mathbf{X}_{i})}} \end{array} $$

where **X**_*i*_ is a matrix that holds the covariates for all the units *j* of child *i*, **X**_*i*_**b** is the fixed part of the model, and var (*y*_*ji*_|**X**_*i*_) is the heteroskedastic conditional variance of the responses, equal to the conditional variance of the total residual of growth model 1: $var(\beta _{i0})+age_{ji}^{2}*var(\beta _{i1})+2age_{ji}*cov(\beta _{i0},\beta _{i1})+var(\epsilon _{ji})$, where *β*_*i*0_ denotes the random intercept, *β*_*i*1_ the random slope, and *ε*_*ji*_ the level-1 residual. Assuming that the response is Normally distributed across all ages, the percentile charts were based on centile values calculated for each age as:

7 $$\begin{array}{@{}rcl@{}} C_{age}=\mathbf{X}_{i}\mathbf{b}+K*\sqrt{var(y_{ji}|\mathbf{X}_{i})} \end{array} $$

where *K* is a value from the inverse cumulative Standard Normal distribution: for example, for an age-dependent centile value along the 25^*th*^ percentile *K*=−0.68. In order to present the charts on the original scale, equation 7 was back-transformed using: (*C*_*age*_×*λ*+1)^1/*λ*^.

In an attempt to estimate the precision of the individual percentiles we used bootstrapping. We chose to obtain the bootstrapped standard error for the median, the interquartile range percentiles, and the 3^*rd*^ and 97^*th*^ percentiles that lie on the extreme ends of the cumulative distribution. Each one of these percentiles was estimated for a given age. Three ages were chosen based on the 25^*th*^, 50^*th*^, and 75^*th*^ percentiles of the age distribution of the intensively assessed sample and correspond to a 74-, 93- and 127-month old child. The original dataset was bootstrapped 500 times. The weights and the *λ* parameter of the Box-Cox transformation were adapted to each bootstrapped dataset and each resample used a new set of weights and a new value for *λ*.

***Application results***

**Fig. 3:**
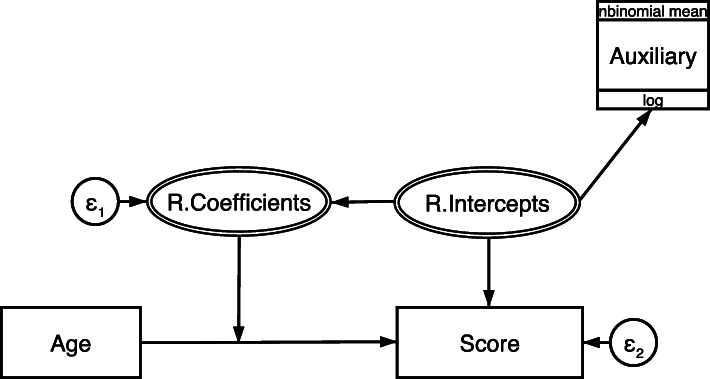
The negative binomial auxiliary model. Boxes represent observed variables and circles represent latent variables. As in a typical growth model, the continuous variable Age affects the partially observed repeated measurements language score variable. The random intercepts factor, which represents current ability, is predicting the responses, the auxiliary variable and the random coefficients factor which represents the individual velocities. The association between the two factors is captured by allowing the random intercepts to predict the random coefficients

While fitting the Poisson auxiliary model, we found evidence of over-dispersion in the CCC-S score, which violates the equi-dispersion assumption of the Poisson distribution. The BIC values from the Poisson and the negative binomial auxiliary models were 49682.55 and 48292.4, respectively. As a result, all subsequent analyses of the SCALES data utilise the negative binomial auxiliary model shown in Fig. 3.

In Table 5, we present the parameter values that were obtained after fitting the weighted model and the negative binomial auxiliary model (NbAux) to scores from the Expressive Vocabulary test. Age is in months and is centred at its mean from the first wave of data so that the fixed intercept corresponds to an expected Expressive Vocabulary score of a 72-month old child. NbAux estimates this value to be 30 whereas the weighted model 33. The variance of the Expressive Vocabulary scores at age 72 months is higher for the NbAux model than for the weighted model, but the variance of the individual velocities appears very similar. Both models estimate a positive correlation between current ability and individual velocities, indicating that higher Expressive Vocabulary scores at age 72 months are generally associated with higher rates of improvement.

**Table 5:** Growth parameter estimates from the growth model that uses weights and from the negative binomial auxiliary model (NbAux) that utilises the auxiliary variable CCC-S

	Weighted model coef. (st.error)	NbAux coef. (st.error)
Fixed intercept	33.3329 (0.5661)	29.8971 (0.4674)
Fixed Age (months)	0.7078 (0.0161)	0.6751 (0.0119)
Variance of current ability	65.1521 (6.8122)	70.5858 (6.5967)
Variance of individual velocities	0.0368 (0.0059)	0.0336 (0.0042)
Current ability and velocities covariance	0.4432 (0.1753)	0.5431 (0.1192)
Level-1 residual	35.2039 (4.2362)	38.2550 (2.6831)

Both models were fitted to 1,289 observations from the Expressive Vocabulary test. The NbAux model utilised an additional 6,411 observations from the CCC-S test

**Fig. 4:**
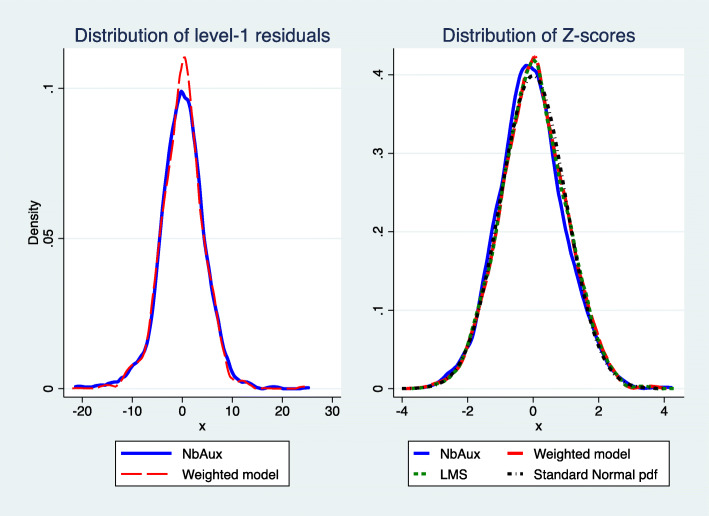
Model residuals (left) and Expressive Vocabulary Z-scores (right) as calculated by parameters of the negative binomial auxiliary model (NBAM), weighted, and LMS models based on 1,289 observations. The theoretical shape of the Standard Normal distribution is also superimposed (Standard Normal probability density function (pdf))

The distribution of the (level-1) residuals from the weighted and the negative binomial auxiliary models are displayed in the left panel of Fig. 4. The right panel of Fig. 4 displays distributions of Expressive Vocabulary Z-scores, calculated from the negative binomial auxiliary model, the weighted model, and the LMS method. It can be seen that while the distribution of the residuals from the weighted model exhibit a sharper peak relative to that from the negative binomial auxiliary model, the resemblance of the Z-score distributions across the models in the right panel is striking and in agreement with the theoretical probability density function of the Standard Normal distribution.

**Fig. 5:**
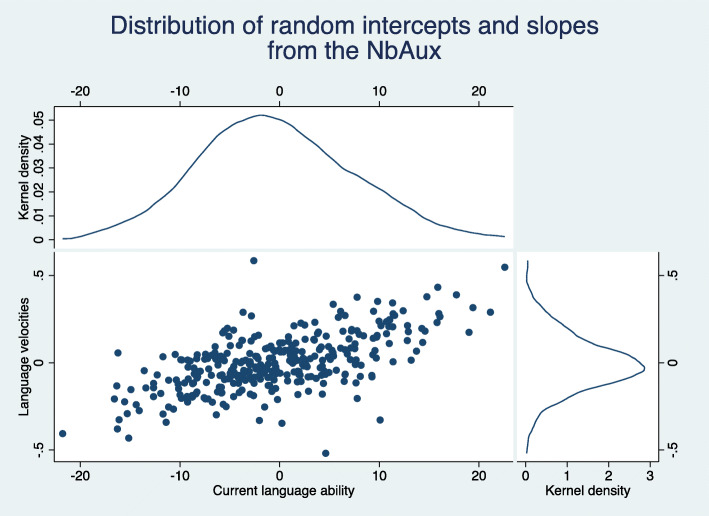
Scatterplot and histograms of predicted random intercepts and slopes from the negative binomial auxiliary model (NBAM) that utilises the auxiliary variable CCC-S

**Fig. 6:**
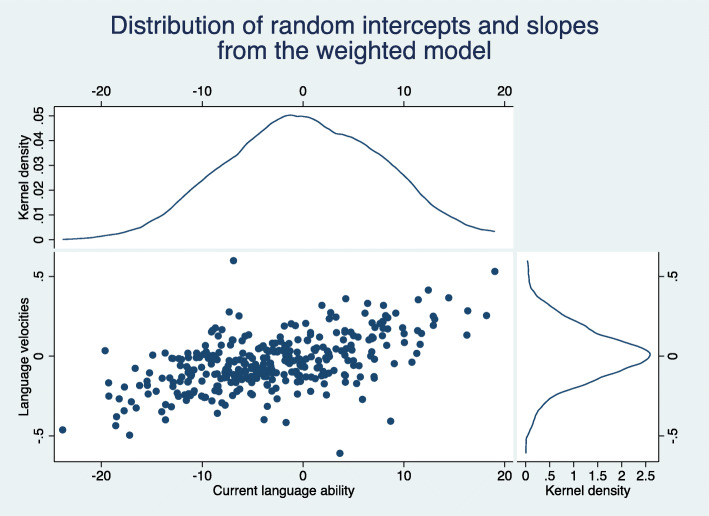
Scatterplot and histograms of predicted random intercepts and slopes from the Weighted model

Figure 5 shows the bivariate distributions of the predicted current language ability and language velocities produced by the negative binomial auxiliary model. For comparison, an analogous graph was created for the weighted model. This can be seen in Fig. 6. The distributions from the negative binomial auxiliary model look Normal although the distribution of the predicted velocities is not centred entirely around zero. This is because the graph includes children who have an outcome observation at the final time-point only. If we were to include all children in the graph we would end up with a large number of children for whom we had no data other than their CCC-S scores. Having incomplete data, the empirical Bayes estimates for these children are naturally *shrunk* towards the mean of their prior distribution, which is zero.

**Fig. 7:**
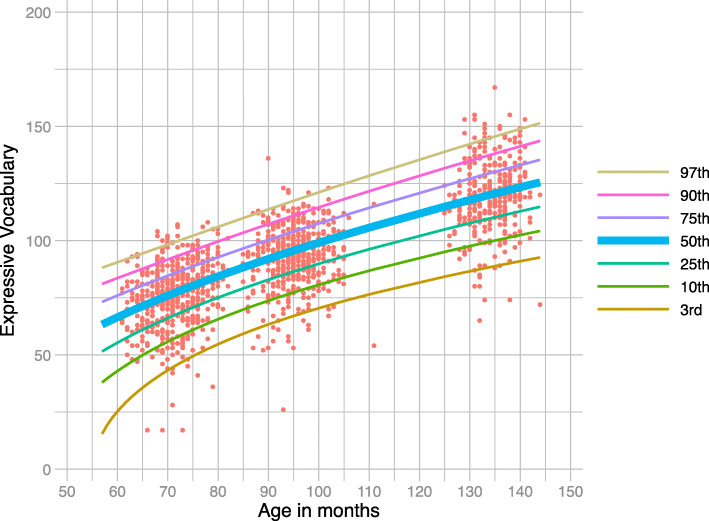
Centile-curves for the Expressive Vocabulary test, based on the whole population (n=6,411). The bold curve in the middle depicts median centile values across age. The thin lines represent the 3rd, 10th, 25th, 75th, 90th and 97th centile-curves

Figure 7 displays the distance chart for the Expressive Vocabulary test based on parameter values from the negative binomial auxiliary model shown in Table 5. It can be seen that the general trend shifts upwards, demonstrating that vocabulary improves with age, as expected. To be placed on the 75^*th*^ percentile, a 90-month old child is expected to achieve a score of about 100 in the Expressive Vocabulary test, and then again a score of about 125 if they wish to maintain the same position on the chart 35 months later. A more subtle feature of the chart is that the curves are not equidistant nor are they parallel to each other; for example, the gap between the 3^*rd*^ and the 10^*th*^ centile-curves is bigger than the gap between the 90^*th*^ and the 97^*th*^ percentiles. This makes the child’s improvement required to maintain their population percentile, in terms of vocabulary growth, dependent on their initial placement on the chart.

**Fig. 8:**
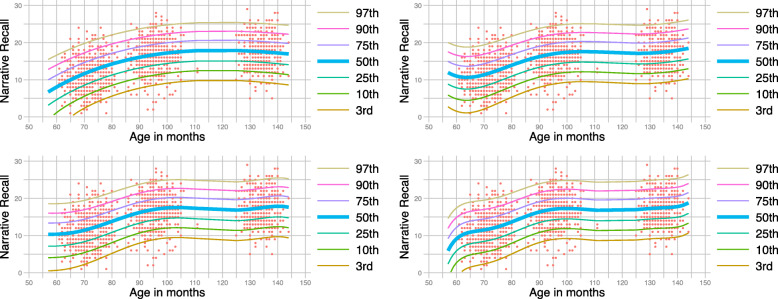
Centile-curves for the Narrative Recall test based on the whole population (n=6,411). The bold curve in the middle depicts median centile values across age. The thin lines are the 3rd, 10th, 25th, 75th, 90th and 97th centile-curves. The top left panel represents a 3^*nd*^ degree polynomial in age. The top-right, bottom-left and bottom-right panels use a 5^*th*^, a 6^*th*^, and a 7^*th*^ degree polynomial, respectively

In Fig. 8 we illustrate the ability of the growth model to adapt to the data patterns in a similar fashion to the way the ‘tuning’ parameters, L, M, and S control the smoothness of the curves in the LMS method (see Cole and Green [3] for details). Here, data come from the Narrative Recall test. All panels of Fig. 8 are built by the negative binomial auxiliary model. The top left panel used a 3^*rd*^ degree polynomial in age, the top-right panel used a 5^*th*^ degree polynomial, the bottom-left panel a 6^*th*^ degree polynomial, and the bottom-right panel a 7^*th*^ degree polynomial. It can be seen that the higher the degree of the polynomial, the less smooth the lines becomes, in an analogous way to the LMS method. Quite often, the choice regarding the level of smoothness is made by eye. This, however, can be aided by formal tests. Since the models here are nested, the likelihood ratio test can be used to strengthen our choice. A comparison between the likelihood of the models that used a 3^*rd*^ and a 5^*th*^ degree polynomial favoured the model with the 5^*th*^ polynomial (p <0.001). The likelihood tests between the models that contained a 5^*th*^ and a 6^*th*^ degree polynomial and those that contained a 5^*th*^ and a 7^*th*^ degree polynomial were not significant (p=0.4153 and p=0.0989, respectively) pointing towards a preference for the 5^*th*^ polynomial. Regarding goodness-of-fit, it is worth noting that after examination, the distributions of the level-1 and level-2 (predicted random effects) residuals were almost impervious to the choice of a polynomial power.

Table 6 shows the results from the bootstrap exercise. Besides the 3^*rd*^ percentile of a 74 month old child, the bootstrapped standard errors returned by the weighted model are consistently larger than those returned by the negative binomial auxiliary model (NbAux).

**Table 6:** Standard errors from 500 bootstrapped datasets for the weighted and the negative binomial auxiliary (NbAux) models, by age and percentile

	Percentile	Weighted model Observed coef.	Weighted model Standard error	NbAux Observed coef.	NbAux Standard error
127 month old	97th	146.1794	1.493828	143.6447	1.404568
	75th	129.0548	.894502	125.0589	.7733639
	50th	119.6728	.813757	114.9004	.6862632
	25th	110.4371	.9443838	104.9204	.8322449
	3rd	94.30761	1.428025	87.54849	1.346387
93 month old	97th	120.0104	.9901348	118.3175	.8779767
	75th	104.4008	.6579751	100.753	.5575749
	50th	95.87027	.6524922	91.18928	.5381976
	25th	87.49048	.7577963	81.82425	.6444512
	3rd	72.90685	1.084865	65.61384	1.051341
74 month old	97th	107.284	.9212999	105.8401	.7348498
	75th	91.59992	.6564309	88.07831	.5309773
	50th	83.0562	.6694443	78.44596	.5517361
	25th	74.68662	.7841305	69.04718	.7010522
	3rd	60.18923	1.153478	52.8808	1.243136

## Discussion

Generally, the objective of statistical analyses is to make inferences that apply to the population targeted by the complete sample. With stratified surveys, where data are mainly missing by design, the aim remains the same but the methodology is complicated by the requirement to compensate for the missing data. This is because if selection into the sample depends on the values of certain variables, which can be related to the response variable, the distribution of the sample data can be very different from the distribution which holds in the population if these variables do not inform the statistical model in some way.

Typically, weighted estimators have been used in the analysis of surveys. The basic idea behind weighting is that by considering a weighted unit as representative of a number of population units, then the sum of all weights equals the size of the target population, and the distribution of the weighted data match the distribution of the population. The closer the weighted unit resembles the population units the closer the matching of the two distributions. Weighted estimation, however, is inefficient and its use in multi-level modelling hampered by lack of clear methodology at the lowest level of data. Incorporation of Normally distributed auxiliary variables into maximum likelihood estimators has been shown in the literature to be as unbiased and more efficient than weighted estimation. Broadly, auxiliary variables are (fully observed) variables that hold information on the study design (or equivalently, on the missingness mechanism), and are related to the (partially observed) response variable of interest. Use of non-Normal auxiliary information in studies that suffer from “design missingness” is under-explored.

To explore the effect of auxiliary information on the estimation of model parameters when data are missing by design, we created artificial data that mimic the two-stage design of the SCALES population survey. We simulated Poisson data to serve as our auxiliary variable, copying the role of CCC-S in SCALES. Then, we simulated repeated measurements of a Normally distributed response and deleted approximately half of it. Deletion of data was based on the auxiliary variable. We tested a number of growth models that incorporate information on the study design either in the form of weights or in the form of an additional variable. The models that contained the additional variable, were structural equation models that were primarily made up of an equation for the growth model and an equation for the additional, or auxiliary, variable. The auxiliary variable was predicted by a random intercept factor and was fitted assuming either a Poisson or a negative binomial distribution, arguably the two most common choices for the analysis of count data. In an extra set of structural equation models we added an equation where the probability of a value being missing was predicted by a constant and the random intercept factor. We also fitted the two structural equation models pretending the auxiliary variable was Normally distributed after a skew-minimising transformation.

The results of our simulation study showed that the best performing models were the Poisson and the negative binomial auxiliary models shown in equation 2 and in Fig. 3, respectively. Our results showed that none of the parameters estimated by the two models contained any significant bias. The standard errors associated with each examined parameter were on average very close to their corresponding empirical standard deviation indicating the lack of bias in the estimation of the standard errors. In terms of coverage, all rates were close to the nominal level.

No significant biases were detected with the weighted growth model during parameter estimation. In line with the literature, our simulation results, too, showed a reduction in the efficiency of the parameters when compared to the best performing auxiliary models. For the random coefficients parameter, the weighted model produced the largest, on average, standard errors among the contested models. We found that the standard error for the fixed intercept parameter was over-estimated compared to its empirical standard deviation by 8.5% on average, the largest percentage difference observed for this parameter. Coverage rates were all very close to the nominal level.

None of the other models fared well in terms of bias, efficiency and coverage; coverage dropped considerably for some biased parameters. The models that included the probability of missingness as an extra equation performed, at times, even worse than the naive model, which did not include any auxiliary information even though it was fitted on incomplete data. Although Normality was not achieved after transforming the auxiliary variable, it was nonetheless used as the outcome of simple linear regression. The bad performance of the models that made use of the transformed auxiliary variable reveals the pernicious effects of model misspecification and highlights the necessity to respect the distributional assumptions about the data generating mechanism.

We also presented an application of our best performing model on actual SCALES data. We deployed language test scores to estimate model parameters, used these as a means to compare the method to established statistical techniques for the analysis of survey data, assess model fit, and produce distance norms and charts.

To this end, we used the negative binomial auxiliary variable model and compared it primarily with a weighted growth model. We chose the negative binomial distribution to model our auxiliary variable because it fitted our data better. The results from this section showed that model fit (assessed through the graphical comparison of the distributions of the residuals and the predicted random effects) for the auxiliary variable model appears as satisfactory as its weighted competitor and that both models produce very similar empirical growth parameters. In terms of Z-scores, our model produced very similar values to LMS, one of the most popular methods for the production of standardised scores. We also provided evidence, after conducting a bootstrap exercise, that use of an auxiliary variable yields more efficient percentile norms than the use of weights, especially in regions of the distribution where the main bulk of the data lies.

### Limitations

Variables can be used as auxiliary variables if they are observed for the whole population targeted by the analysis, and are highly correlated with the incomplete variable of interest. As such, these variables are often not available or are themselves fraught with missing values, making the MAR assumption about the data less likely to be met. In SCALES, the CCC-S questionnaire was the dominant selection criterion, and hence a single auxiliary variable was sufficient. When there is more than one selection criteria, use of multiple auxiliary variables can make model estimation cumbersome. On these occasions, a weighted approach may be preferable since weights can easily allow for complex sample selection.

## Conclusions

In this study, we compared an auxiliary variable model with a model that utilises weights in terms of bias and efficiency of their estimators. Both methods can easily incorporate information on the sampling design of the study and help retrieve lost information. We chose to test a Poisson distributed variable as auxiliary and showed how easily this variable can be incorporated into a structural equation model. We found that the auxiliary model is not only as unbiased as its weighted counterpart, it also offers additional gains in efficiency.

Whenever plausible, we recommend the use of auxiliary variables over the use of weights. Besides yielding estimates with reduced efficiency, the weights are not well suited to longitudinal data with incomplete and only partially overlapping waves. No mainstream method allows for the use of weights at the response level, and researchers who wish to conduct longitudinal analyses resort to complicated methods regarding how best to handle the different missing data mechanisms operating at different time points. The use of auxiliary variables does not suffer from this.

The ability of our model to use not only the Poisson but also the negative binomial distribution makes our method amenable to a wide range of disciplines, such as psychopathology and chronic disease, in which overdispersed counts arise very commonly from screening questionnaires.

The auxiliary variable approach also has an advantage over the LMS method, in that it can estimate, as well as population percentiles, velocities that we can make additional use of, for example, in assessing the speed of a child’s language or cognitive improvement.

We finally saw how parameters from the auxiliary variable model can readily be used not only for estimation and inference but also for the production of growth charts without the need to use weights.

In this study, we tested the effect of one discrete auxiliary variable. Future research can test the estimation performance of models that host more than one auxiliary variable. This would be beneficial, for example, in surveys where more than one selection criterion exists. The extra auxiliary variables can be incorporated either as predictors of the random intercept factor or form responses in additional equations similar to those used in this paper. Finally, the effects of different types of auxiliary data can also be explored. Here we chose to work with count data due to SCALES. Testing the estimation performance of models after the incorporation of binary auxiliary data, for example, would be of great interest.

## Data Availability

This work used simulated data. The Application section used data from the SCALES study and an anonymised data file has been uploaded to the journal alongside the manuscript. For any further data requests/questions please contact the corresponding author. All methods were carried out in accordance with relevant guidelines and regulations.
